# The Vagabond Sage

**Published:** 1881-08

**Authors:** 


### THE VAGABOND SAGE.

An old man of very active physiognomy was brought to the police court. His clothes looked as if they might have been bought second-hand in his youthful prime, for they had suffered more from the rubs of the world than the proprietor himself.

"What business?"

"None; I'm a traveler."

"A vagabond, perhaps?"

"You are not far wrong. Travelers and vagabonds are about the same thing. The difference is that the latter travels without money and the former without brains."

"Where have you traveled?"

"All over the continent."

"For what purpose?"

"Observation."

"What have you observed?"

"A little to commend, much to censure and a great deal to laugh at."

"Humph! What do you commend?"

"A handsome woman that will stay at home, an eloquent preacher that will preach short sermons, a good writer that will not write too much and a fool that has sense enough to hold his tongue."

"What do you censure?"

"A man that marries a girl for her fine clothing, a youth who studies medicine while he has the use of his hands and the people who will elect a drunkard to office."

"What do you laugh at?"

"I laugh at a man who expects his position to command that respect which his personal qualifications and qualities do not merit."

He was dismissed.—*Exchange.*

---

Flies Tormenting Horses.—Take 3 small handfuls of walnut leaves, upon which pour about 3 quarts of cold water; let it infuse over night, and the next morning pour into a kettle and let it boil for about fifteen minutes. Use cold. Moisten a sponge with the preparation, and before you take the animal out of the stable, wash him between and upon the ears, the neck, flanks, etc.

Young lady examining some bridal-veils—"Can you really recommend this one?" Over zealous shopman—"Oh! yes, miss. It may be used several times."

**LIFE AND DEATH.**—One half the human family dies under seventeen years of age. Nine tenths of all who are born ought to complete their "three-score years and ten," because nine tenths of all diseases are avoidable by the steady practice of temperance and such out-door activities as are encouragingly remunerative. There is a still more specific method of lengthening life in healthfulness and vigor, and one which is practicable by the masses. Colds or constipation immediately precede or attend almost every case of ordinary disease. The latter can be antagonized by abstinence, cleanliness, and warmth for thirty-six hours; and a cold need not be taken once a year if three things are attended to. Avoid chilliness, damp clothing, and cooling off too soon after exercise.

